# Improving long-term care provision: towards demand-based care by means of modularity

**DOI:** 10.1186/1472-6963-10-278

**Published:** 2010-09-21

**Authors:** Carolien de Blok, Katrien Luijkx, Bert Meijboom, Jos Schols

**Affiliations:** 1 Amsterdam Centre for Service Innovation (AMSI), University of Amsterdam, the Netherlands; 2 Department of Tranzo, Tilburg University, the Netherlands; 3 Department of Organization and Strategy, Tilburg University, the Netherlands; 4 Department of General Practice, Maastricht University, the Netherlands

## Abstract

**Background:**

As in most fields of health care, societal and political changes encourage suppliers of long-term care to put their clients at the center of care and service provision and become more responsive towards client needs and requirements. However, the diverse, multiple and dynamic nature of demand for long-term care complicates the movement towards demand-based care provision. This paper aims to advance long-term care practice and, to that end, examines the application of modularity. This concept is recognized in a wide range of product and service settings for its ability to design demand-based products and processes.

**Methods:**

Starting from the basic dimensions of modularity, we use qualitative research to explore the use and application of modularity principles in the current working practices and processes of four organizations in the field of long-term care for the elderly. In-depth semi-structured interviews were conducted with 38 key informants and triangulated with document research and observation. Data was analyzed thematically by means of coding and subsequent exploration of patterns. Data analysis was facilitated by qualitative analysis software.

**Results:**

Our data suggest that a modular setup of supply is employed in the arrangement of care and service supply and assists providers of long-term care in providing their clients with choice options and variation. In addition, modularization of the needs assessment and package specification process allows the case organizations to manage client involvement but still provide customized packages of care and services.

**Conclusion:**

The adequate setup of an organization's supply and its specification phase activities are indispensible for long-term care providers who aim to do better in terms of quality and efficiency. Moreover, long-term care providers could benefit from joint provision of care and services by means of modular working teams. Based upon our findings, we are able to elaborate on how to further enable demand-based provision of long-term care by means of modularity.

## Background

Putting the health care client rather than the care supplier at the center of processes and structures has been advocated by several proposals on future care provision [e.g. [1-3]]. Hence, the needs and expectations of patients and clients are now being viewed as the starting point in a thorough re-orientation of roles, tasks, operational processes, organizational structures, and inter-organizational cooperation in the promotion of a demand-based approach [2]. More specifically, redesigns have been developed, such as clinical or care pathways, focused factories, and integrated care [4-6] that treat clients in a specific, predefined care trajectory. A characteristic of these new designs is that they have often been developed for a particular and well-defined client or patient group with relatively simple demands for care concerning only one constraint or disease [7]. As a result, care delivery for these clients is becoming more demand-based, without sacrificing too much efficiency and cost containment, which are other pressing factors that care providers must take into account in day-to-day care provision [8].

In the light of our aging societies, however, the nature of demand for care is subject to change. Population aging results largely in the growth of chronic and long-term conditions. Especially for the elderly, these conditions are often accompanied by multiple, diverse and non-linear needs for different types of care and related services [9,10]. Elderly clients often have multiple demands in various aspects of life, such as health, welfare, housing, transportation, and support [e.g. [11,12]], because they want to live independently as long as possible. Besides, we cannot speak of *the *needs and wants of *the *elderly [13]. Diversity among elderly can be expected, on the one hand, because of differences in the health status and medical requirements of individual clients [10,14]; an elderly client with diabetes has different needs from an elderly client who suffers from problems with bending and stretching. On the other hand, the elderly differ from each other in personality, background and lifestyle, which is likely to cause the content of their needs and desires to differ as well [13]. Furthermore, needs and requirements are likely to vary over an individual's life course as a result of changing health conditions. Because health, generally speaking, deteriorates over time [e.g. [10]] the elderly will require more intensive care and services. Moreover, new constraints are likely to arise with age, thereby increasing the prevalence of multi-morbidity - the simultaneous occurrence of various (chronic) conditions [15,16], causing the elderly to require a wider spectrum of care and services over time.

The complex and heterogeneous needs and requirements for chronic and long-term care can hardly be answered in well-delineated, unidirectional, and efficiency-driven care processes, as developed in other health care settings [4-6]. Care and services offered through these processes will be likely to have too narrow a focus to accommodate widely varying needs. In addition, the existing processes hamper a holistic view of the individual elderly client as well as coordination across the various ailments from which a client might suffer [7]. Therefore, the long-term care sector is in need of new approaches and visions that enable the sector to improve care delivery towards the development of demand-based care, and simultaneously accommodate the complex nature of demand [17,18]. In this respect, Hofmarcher et al. [7] propose to go beyond a re-orientation of care delivery *processes *and focus on redesign of the health *system *architecture as a whole. This means that long-term care providers should organize their *processes *as well as the *care and service parts *offered and provided and *professionals *involved in care provision in line with underlying principles of what they want the care system to achieve. To support the development of a demand-based care system, four dimensions should be taken into account simultaneously [19]:

• choice options,

• variation,

• client interaction, and

• joint delivery.

By increasing the available range of choice options, it becomes more likely that clients will find care and service parts that optimally suit their particular circumstances. In addition, it should be possible to combine all options provided in any way desired by the client. Combining care and service parts available into differing configurations creates variation in the final offering across clients with diverse needs. Furthermore, client interaction should be stimulated and managed during the care process in order to consult clients on their needs for care and be able to adapt and customize the care offered accordingly. Finally, whereas many care organizations and even organizational departments work according to autonomous and separate processes and structures [20], demand-based care provision implies that organizations jointly take care of a client's multiple demands and serve the client in an integrated fashion. Following these four dimensions, demand-based provision of long-term care to the elderly would then imply that care and service parts ranging from dissimilar and heterogeneous areas of life, and possibly from different organizations, are combined into a single packet offered that is in turn customized, in cooperation with the client, to the individual's needs and wants [19].

Conceptually speaking, the four dimensions identified can be answered by means of modularity. Modularity is a concept that stems from the field of operations management and is increasingly recognized in health care as a means to design client-centered products and processes [e.g. [8,19,21-25]]. Moreover, the concept of modularity facilitates an efficient way of working, which suits the pressure for cost containment that is faced by many providers of long-term care [26,27]. These features drive us to further investigate the potential of modularity in moving the sector of long-term care for the elderly towards the objective of demand-based care provision and underpin the practical and theoretical relevance of this study. The two main purposes of this study are then are the following: (1) evaluate current long-term care provision from a modularity perspective with respect to the four dimensions related to demand-based care, and (2) identify remaining gaps in demand-based care provision and discuss how to further enable demand-based provision of long-term care by means of modularity principles and practices. To this end, we will elaborate upon the concept of modularity in the next section. Thereafter, the methodology for our empirical research is given. Finally, the findings are presented, discussed, and elaborated upon in the light of modularity theory.

### Theoretical background

#### Lessons from modularity

In one of the first contributions to the literature on modular production, Starr [28] formulates the basic idea behind modularity as ''... design, develop, and produce [...] parts which can be combined in the maximum number of ways''. As such, modularity proposes to create a custom product or service from standardized components [e.g. [29-31]]. Modular production principles traditionally stem from a manufacturing setting and have not been specifically developed for services or health care. However, the attention given to modularity in a wide range of service and care settings is growing [e.g. [8,32-34]]. For example, modularity is applicable to elective surgery to deal with heterogeneous patient needs, since the set of standardized operations selected and combined for the surgical treatment may differ from one patient to another [8].

There are several important dimensions related to the concept of modularity: components, modules, interfaces, and packages. *Components *are parts that perform one clearly defined function in a final product or service offering. They are the smallest units into which a product or service can be divided [e.g. [33]]. In care, a wide variety of components can be discerned such as cleaning the house, washing, assistance with getting dressed, insulin injection, meal service, taxi transportation, financial advice, etc. A *module *is understood as a conceptual grouping of one or several components that provide variants and substitutes to the same functionality [e.g. [33]]. In care, components can be grouped into modules such as care services, welfare services, safety services, and housing services. *Interfaces *are linkages shared among components. In general, they manage the interactions and connections of components when they are combined into a final packet offered [30,34]. In care, protocols, procedures, and standard lines of communication ensure that the selection of components to be provided to a single client make up a united and coherent whole. Combining and connecting various components by means of interfaces creates a modular packet offered [35]. We will refer to these as modular *packages*.

The main aim of modularity is to allow organizations to mix and match components into modular packages that meet closely the diverse customer preferences [8,36]. However, modular packages have to differ only with respect to those components where customers have different requirements. As such, not all components need to be subject to change to address market heterogeneity [24,30] and similarities among clients can be exploited [13]. In elective surgery, for example, preoperative evaluation and postoperative care are often very similar for all patients. The same holds for certain operations during the surgery process for particular patient groups [8].

Modularity concepts address not just an organization's product or care offerings, but also an organization's working processes and the organizational arrangement of people [33,37]. Process modularity allows for the mixing and matching of process components, or activities, in order to create customized packages with and for clients. A general principle is that standardized activities should be performed first and customization activities should be postponed, occurring later in the process to allow for customization in the most effective manner [37,38]. A modular organizational arrangement implies that an organization's workers are assigned to clearly defined teams or divisions. Each team is responsible for the provision of one or more service components and (members of) the teams can be combined and reorganized according to customer requirements [37].

#### Leads of modularity in the light of demand-based care

With respect to *choice options *and *variation*, Chorpita et al [21] state that modularity allows for the configuration of a large number of therapeutic interventions in psychotherapy to be created from a fairly standardized range of exercise and activity components, such as relaxation, social skills training, and problem solving skills training based on individual client's needs. This allows for greater adaptability of the therapy to different types of patients [28,38,39]. For long-term care, modularity would then imply that every client can be offered a different combination of care and related components and thus each is treated as unique. Moreover, modularity allows for the adaptation of a product or service package over time since component variants can easily be added, substituted, omitted, or modified [40,41]. Building on the psychotherapy example, a particular patient's therapy can be adapted over time by adding new interventions or adapting exercise or activity components [21]. Especially in the sector for long-term care, being characterized by changing client demands, it is crucial that the modular package can be reconfigured continuously to be appropriate to expressed or implied needs.

With respect to *client interaction*, the provision of modular packages in a homecare setting have been found to ease the interaction between client and provider, since all modules and components required by a client could be assessed simultaneously [25]. This resulted in savings in terms of time and effort invested in needs assessment. Moreover, since components form a pre-specified, transparent, and well-organized range of options, customers and professionals can more easily interact when specifying their required combination of different components and/or activity sequences [42]. Regarding long-term care provision, modularization could then facilitate interactive package specification and enable professionals to take into account the client holistically.

Concerning *joint delivery*, Bohmer [8] posits that required modules and components may well originate from multiple providers. This can, for example, be seen in outpatient care for chronic conditions such as hypertension where treatment may include weight control, stress control, diet modification, drug therapy, and on-going surveillance. Each component may be provided by a separate professional or a separate organization but the combination of the components makes the hypertension treatment uniquely suited to each patient [8]. Because a modular set-up of supply uses standardized and well-tuned connections, components provided by different suppliers can be seamlessly combined into one care package. For clients of long-term care, who often require care and services from several suppliers in various fields, this would increase the likelihood that all their needs and requirements will be covered. In summary, despite the newness of the concept, modularity provides leads to bring about the four dimensions related to demand-based care provision in long-term care.

### Research methods

#### Study design

To address the two main purposes of the present study, two research questions were formulated:

RQ1: Which modularity practices are currently used in long-term care provision in order to provide demand-based care?

RQ2: What is the potential of modularity in moving the sector of long-term care for the elderly towards the objective of demand-based care provision?

Given the limited amount of information on care modularity and the exploratory character of the study objectives, a qualitative case study design was used [43,44]. Interviews were used as the main method for data collection since we aimed to get detailed and accurate data on actual working practices. Data collection focused on the current set-up of supply and the process of needs assessment and care package specification (in short, the specification process). In this process, a fit is made between the often ambiguous and complex needs of clients on the one hand, and the care and services available from the long-term care provider on the other hand.

The case study research took place in the context of long-term care provided to elderly living independently where policies promoting care provision based on client needs have been in vogue. To put the client at the center of long-term care provision the Dutch government has introduced laws and regulations that contribute to the empowerment of elderly care clients and the creation of choice in and diversification of supply in care, welfare, and housing [e.g. [45-47]]. In addition, the payment system has been reformed to promote free choice and market competition as well as fair distribution of care.

When an elderly person is in need of long-term care, he or she can receive support through several sources of finance, depending on the type and severity of the individual's needs and requirements. When a client is suffering from severe complaints that concern mainly the provision of care, he or she can apply for funding from the Exceptional Medical Expenses Act (Dutch: AWBZ). This is a law by which every Dutch citizen is ensured of care when suffering from chronic illness, handicaps, or old age complaints and, as such, it regulates and finances care for the elderly [48]. An elderly person can gain access to funding from this act by applying for an indication at what is called, the Central Indication Organ (Dutch: CIZ). This is an independent body that assesses a citizen's needs with respect to several care functions distinguished in the AWBZ, such as personal care or nursing care. When in need of social services, such as home help, housing adaptations, or meal services, an elderly person can apply for an indication under the Social Support Act (Dutch: Wmo) [49]. Once possessing an indication under either act, a client can choose the provider from which he or she wants to receive care and services, and this provider, in turn, can be sure that the care and services provided will be reimbursed by the government. Finally, a part of the care and service range offered by providers has to be paid for or can be insured by the elderly person himself. This concerns primarily luxury and supportive services, such as leisure activities, courses, and dietary advice.

In summary, the financing structure in long-term care is aimed at creating a system in which well-founded needs and requirements of individual elderly clients form the starting point for a process that ultimately leads to the creation of compound care and service packages to be provided jointly by various types of care and service providers.

#### Sampling

The case study research was conducted in the south of the Netherlands. To be able to draw solid conclusions on our rather novel subject we followed a sampling strategy based on literal replication. This means that we selected cases because they demonstrate the same phenomenon under study [50]. To preserve uniformity within our sample and promote meaningful comparison among organizations [51] inclusion criteria for the study were: (1) the organization has elderly clients as its main client population, (2) the organization provides a wide variety of heterogeneous care and service parts, (3) the organization has documented and implemented its product range and processes in an established manner. In addition, we selected for maximum variation [52] to ensure that we covered all types of organizations active in the Dutch sector for long-term care for the elderly. Even though all cases selected provided care and services to elderly people living independently at the time of our data collection, this was a recent development and their background was either in home care or residential care. Since we aim to gain insight into various dimensions related to modularity in the sector as a whole, data collection in both types of organizations was essential for our research.

Five suitable case organizations were identified by means of internet searches and informal networking. In preparation for each case investigation, we mailed letters of invitation to the boards of directors of the five long-term care providers. In a meeting with each board, questions were asked to screen the inclusion criteria and to check their willingness to participate. One organization did not fulfill inclusion criterion 3, i.e. this organization had not documented and implemented its products and processes in an established manner, so the final sample consisted of four organizations. This was enough to satisfy our criteria for maximum variation. Table 1 summarizes the various characteristics of the organizations included in this case research.

**Table 1:** Sample of case organizations

	Case 1	Case 2	Case 3	Case 4
*Main client population*	Elderly clients			

*Service range*	Long-term care, welfare services, domestic services, leisure activities, social support, safety services, comfort services, residential care and services			

*History in*	Home care	Residential care	Home care and residential care (merger)	Home care and residential care (merger)

#### Recruitment

A principle informant was appointed for each case organization. The principle informant held a key position in his/her organization (i.e. close enough to the specification process to know about its specifics and particularities but distant enough to keep an overview of issues related to care provision in the organization as a whole), and therefore was best informed about whom to interview to gain valuable data [53]. This enabled us to select interviewees who could best provide detailed insight into specific working practices regarding the issues being examined. Participants were all related to the subjects under study; however, to gather data that was as comprehensive as possible, they regarded different activities, phases, and organizational levels related to the specification process. This allowed us to look beyond partial and biased views in the data obtained. In each case organization, participants were the regional director, team leader home nursing, team leader home help, front desk employees, start-up nurses, key nurses, and representatives from complementary services, call centers, and marketing. Because the aim of our empirical study was to obtain detailed and factual information on a participant's current working practices, to be combined into a complete, comprehensive, all-encompassing, and in-depth view of the organization's working processes, procedures, service supply, etc., collection of descriptive participant statistics was deemed subordinate. As no health care clients were involved in this study and the data collection concerned organizational practices only, approval from an ethics committee was not required.

#### Data collection

Multiple sources of evidence were used for data collection to facilitate a process of triangulation [54]. The main method of data collection was semi-structured interviews. To guide the data collection, a data collection protocol [55] was formed based on our review of the literature. This protocol contained a topic list and indicative questions [54] with respect to all variables to be addressed; the protocol is summarized in table 2.

**Table 2:** Summary of the data collection protocol

Topic	Indicative questions	Sources of information
*Set-up and organization of components*	• What care and service parts does your organization offer and how are these organized? → Range, types, grouping, arrangement. • To what extent are care and service components standardized? → Possibility for choice, adaptation, fine-tuning	Interviews Documents

*Combining components into packages*	• How does the need assessment take place? → Process, activities involved, aiding devices used, people/departments involved • How is the care and service package subsequently configured? → Process, activities involved, aiding devices used, people/departments involved • How do you achieve or ensure unity within one package? → Among components, among people/departments involved	Interviews Documents Observation

*Role of people*	• How do you deal with differences among elderly clients? • How do you deal with different types of elderly client demands? • How do you deal with changes in elderly client demands over time? • What causes similarities and differences in packages among elderly clients? • What is the role of the elderly client in the specification process? • What is the role of care professionals in the specification process? • How does the interaction between client and professional take place in specification process?	Interviews Observation Documents

*Effectuation of demand-based care on operational level*	• How are choice options provided to elderly clients? • How is variation achieved in the care and service packages? • How do you take care of client involvement? • Which departments and organizations work together for one client, are involved in joint delivery? How does joint delivery take place?	Interviews Documents Observation

A trained interviewer interviewed multiple respondents in all cases. All interviewees approached agreed voluntarily to participate in the interviews. An interview typically lasted from one to two hours in duration. Prior to each interview, the participant was informed of the purpose and objectives of the study and how confidentiality of his or her statements would be protected. Questions from the participant were addressed and prior to each interview informed consent was obtained verbally. During the interviews, participants were asked about issues related to the organization of care and service supply and working processes (e.g. "how is the range of care and service parts arranged" and "what process steps are taken to specify and configure an appropriate care and service package") as well as about issues concerning demand-based care (e.g. "how are choice options provided to elderly clients" and "how do you take care of client involvement") by means of open, non-directive questions.

Interviews were audio-taped and transcribed verbatim for subsequent analysis. The interview texts were sent back to the interviewees after transcription to check the interview contents and identify and clarify misunderstandings [56]. In total, 38 interviews were conducted.

The interviews were complemented by examination of relevant documentation that was provided to us by the principal informant. For each case, we consulted process descriptions, product books, handbooks and other documentation such as project plans and quality manuals that would give objective and additional insights into the organization of processes and product supply. We wrote summaries on each document examined. Finally, each case involved three one-day field visits to observe and experience the working processes. Notes taken during the observation visits were written out shortly after the visit to ensure we were able to capture most of the things that had been observed.

#### Data analysis

Thematic analysis was used in order to generate an in-depth exploration of current working practices from a modularity and demand-based point of view. To explore themes and interactions among these themes, we followed a systematic data reduction process that consisted of the following steps: reading of transcripts, document summaries and observation notes, segmentation of sentences and phrases, codification of text segments, generation of themes and categories, and identification of relationships [52]. Segmentation and coding started from an initial codes list that was developed on the basis of our review of the literature on the generic themes and aspects related to the concepts of modularity and demand-based care. Each transcript, document summary and observation summary was processed independently by two researchers who then compared and discussed their codes until consensus was reached. In total, three researchers were involved in the coding process; two researchers each coded half of the interviews and document and observation summaries, the principal researcher processed all to warrant consistency. To balance the modularity and care perspectives to be integrated in this study, the research team (and thus the coders) consisted of researchers whose backgrounds were in either operations management or health service research. During the coding process the initial codes list was expanded to encompass emerging themes and cover the richness and nuances of the data. When no more new themes emerged from the data and all themes were covered for each of the case organizations, we started the identification of relations and as such related the core concepts of modularity to the characteristics of long-term care and the dimensions of demand-based care provision. To increase the accuracy of the insights and findings, the principle informant of each case organization was consulted regularly during data analysis [53]. To code and manage the data, we used the qualitative analysis software Atlas.ti. Using software leads to more systematic analysis procedures and guards against information processing biases, as such improving the validity and reliability of the study [52,54].

After the data reduction, the data required some further processing to permit drawing of conclusions [52]. Therefore, we arranged the data into organized, compressed assemblies of information in the form of blueprints [57]. As such, we were able to show how we built our insights and findings on the arrangement of care and service supply and the specification process.

### Results: modularity in practice

The next four sections discuss the themes and their relationships revealed by our data analysis with respect to modularity principles and practices that are currently used by the case organizations and the four dimensions of demand-base care. The tables presented in this section provide rich and detailed illuminations on the component setup (Additional file 1: Table S1) and specification process (Additional file 1: Table S2) of the case organizations on which the results are based.

#### Choice options: a modular set-up

To answer the widely varying needs of their clients, and support them in continuing to live independently as long as possible, all cases supplied an increasingly wide range of dissimilar components of care and related services on various aspects of life. In principle, each client could make use of all components provided but the actual use of components was very much guided by the medical and physical constraints and diseases from which an individual suffered. Increasingly, the interviewees found that elderly clients were also asking for components because of their personal interests and preferences, especially in social, entertainment and comfort services. Subsequently, following market demand, the range of services that were complementary to the initial provision of care was developing and growing rapidly.

**Figure 1:**
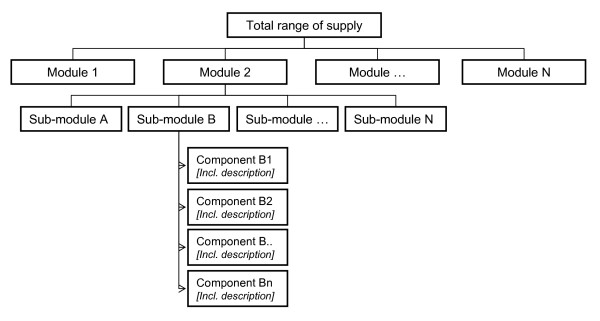
**Structure of the arrangement of care and service supply**.

To manage their widening range of supply, all cases had undertaken the challenge of finding suitable ways to organize their supply under the assumption that a logical set up would create transparency and enable professionals and elderly clients to find and use the options available. Additional file 1: table S1 describes the diversity of approaches taken by the cases in setting up and organizing their range of care and service supply. Even though diverse approaches were used, the outline followed by all cases appeared to be fairly similar. In essence, the empirical data revealed that in each case, care and service modules had been identified that together formed the main building blocks of a menu of choice options. Each module contained a range of sub-modules and subsequent component variations that could complement, supplement or substitute each other. For example, the module 'care' in most cases contained, among others, the sub-module 'personal care' under which components such as 'washing', 'getting dressed' and 'getting ready for bed' were grouped. Components were further specified with respect to e.g. the type of clients, constraints or diseases they for which were meant and possible variations in delivery (e.g. moment, time span, duration, intensity, and location). In each case, the menu as a whole, built from the various (sub) modules, served as a platform from which appropriate components were selected and combined for each individual elderly client. A structure of the arrangement of supply, including modules, sub-modules and components, is depicted in figure 1.

The general arrangement structure used was basically the same for all cases, however, the way in which each case organization elaborated upon the general approach taken differed (Additional file 1: Table S1). Our data indicated that components were grouped with respect to their functionality. In addition, our data revealed other ways of reorganizing components; we observed component groupings that took the client, the professional or combinations of functionality and client as their starting point.

#### Variation: pre-combination as a starting point

In all cases, the range of supply was separated into components. These components in turn could be recombined into any combination required by the organization's elderly clients. As such, components in personal care could be combined with meal service, a computer course, financial advice, and dietary advice, etc. The combination of components that would ultimately be delivered to the elderly client was totally dependent on the individual's situation.

As a device to aid package specification for individual elderly clients, cases made use of predefined base packages. Base packages contained a selection of pre-grouped components that are key to answering the common needs in a particular segment. Diversity among segments of elderly clients was used as a steering principle for the configuration of various base packages. For example, the case organizations developed base packages for the vital elderly, the elderly who needed some assistance in daily activities, the elderly who wanted to live independently but in a sheltered environment, and the elderly in their end-of-life phase. Subsequently, commonalities among elderly clients within one group were used to cluster key components in a base package. For example, in case 2 a base package for elderly in need of some assistance was built around key components concerning homecare activities, social activities, and an alarm service.

The interviewees indicated that the base packages developed by the cases mainly provided guidance in the development of an appropriate package for each elderly client. As such, the packages linked the organization's total range of supply to the process of needs assessment and package specification. The base packages, however, were by no means strict and closed entities, but provided formats based on which individual care and service packages could be further adapted, specified and fine-tuned during the specification process, to be discussed in the next section.

#### Client interaction: managing involvement during package specification

**Figure 2:**
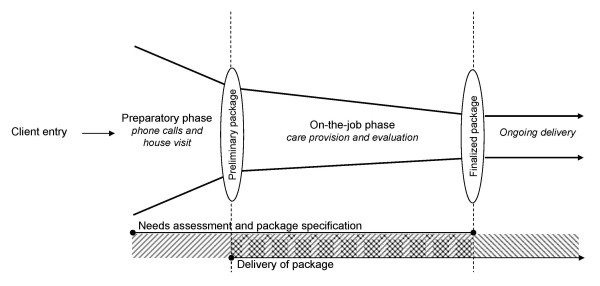
**Specification process**.

In each case organization, the total range of supply grouped into (sub) modules, together with the pre-grouped base packages formed the basis for the configuration of care and service packages. Since all case organizations had the aim of putting the demand of the elderly client at the center of care and service provision, package specification and configuration were based on a thorough needs assessment. In order to meet a given elderly client's demands for long-term care, needs assessment in each case organization was performed in close cooperation between client and professionals. Involvement of the elderly client in the assessment and specification process, thus, was seen as something highly desirable that needed to be stimulated. At the same time, the case organizations were confronted with a downside of client involvement, i.e. the introduction of a great deal of uncertainty and variability in the specification process, which posed the need to control client interaction as well. The set-up of the specification process is described in Additional file 1: Table S2 and depicted in figure 2.

From the description of the specification process, it becomes clear that all cases worked according to a process consisting of two separate, but closely related phases. This set up of the process allowed the cases to manage the complex activity of needs assessment and package specification as well as the degree of client involvement in the process.

In the first phase of the specification process, process steps and activities undertaken were the same for all elderly clients or for the elderly in a particular segment. Even though the exact content of each activity or step might differ, its common structure reduced complexity and helped professionals to overcome the challenge of approaching each client situation as if it were being dealt with for the first time. The second phase of the specification process mainly consisted of activities that dealt with specific client situations. It ensured that the final care and service offered optimally suited an individual elderly client, both in terms of medical constraints and in terms of personality and lifestyle. Care and service packages configured or selected in the first phase were further adapted to individual elderly clients by making use of the menu of options; components were added, intensified, or omitted, as such translating specific client needs into appropriate supply.

The activities that took place in the specification process were, thus, sequenced in such a way that common-to-all activities occurred early in the process while customization activities were postponed until later in the process. Thereby, the first process phase allowed for quick diagnosis and assessment and thereby also a quick start of care delivery to each elderly client. Postponement of customization activities provided time to the case organizations to reveal detailed client specifications regarding their multiple and changing needs and requirements. In this way it was ensured that each elderly client received all the care and related service parts that answered his ailments and/or old age constraints and that these parts were delivered in the client's preferred manner.

Moreover, the case data indicated that process steps were organized according to the intensity of client involvement. Early in the process, interaction with an elderly client was needed to know what kind of needs and requirements had to be answered by the organization. However, the intensity of the client interaction was as low as possible (phone conversation, house visit). As such, the elderly client could not affect the course of the process too much, which allowed for efficient and effective execution of process activities. The intensity of the interaction was increased when activities were taking place to customize an individual's care and service package. As such, client induced uncertainty was centralized in a particular part of the specification process.

#### Joint delivery: a challenge for the future?

In all cases, our data revealed the existence of a well-arranged range of supply and specification processes. Despite this, there was no single, integrated access to all the care and service components available within each organization. This finding held for all cases. Individual divisions and cooperating organizations still very much tended to set up their own care and service packages on offer as well as their own specific (sub) specification lines. If elderly clients required components from multiple divisions of one organization, or of cooperating care and service providers, they often ended up contacting each of these divisions or organizations separately to acquire information as well as access to the services.

Inter and intra-organizational partitions, thus, were highly prevalent. Because of the non-transparency in supply and fragmented accessibility across case departments and organizations, professionals indicated that they were often unaware of the full range of care and service components available. This issue became more prevalent as the case organizations grew larger and expanded their range of care and services to areas that were formerly unrelated to their original core business, i.e. 'pure' care provision. As a result, care and service packages specified for individual elderly clients were often too narrow compared to the multiple needs of the clients and the abilities of the (cooperating) organization. Even though all interviewees in all cases were aware of the existing intra and inter-organizational boundaries, and improvement projects were started to overcome these, joint delivery of care and services both within and across organizations was indicated as being a challenge for the future at the time of our data collection.

## Discussion: Towards modular care provision

Modularity is increasingly recognized in various health care settings as a means to cope with heterogeneous client demands and design care systems that are centered on the health care client [e.g. [8,19,21-25]]. In order to advance the sector for long-term care for the elderly towards its goal of demand-based care provision, we evaluated current practice in this sector through a modularity lens. As such, our study added empirical evidence on the use of different modularity principles and practices in this sector as well as remaining gaps, primarily in relation to joint delivery. In this section, we will discuss our findings in the light of modularity theory.

Moreover, we indicate how long-term care providers could advance the four dimensions of demand-based care by drawing modularity principles and practices further into the operational set up of their organizations. In this respect it is important to stress the dependencies within the organizational system (i.e. the organization's products (services), processes and people). The basis for providing choice options and variation is given by the arrangement of an organization's product (service) supply. However, a well thought-out process is needed in which this supply can be configured into a package based on client needs and requirements. People link products and processes together since service workers and health care professionals are the ones who execute process activities to configure the required product components. Thus, to create a care delivery system that enables service to clients being as demand-based as possible, the organization's product (service) supply, processes and people should be kept aligned.

### Choice options in care service supply

In response to the diversity and multiplicity of demand for long-term care, the case organizations in this study offer an increasingly wide range of care and service supply to provide options and variety. Even though a wide range of services may allow providers to respond to a wide range of needs [58], the provision of choice usually comes at some cost in terms of increased complexity and reduced transparency [59]. In this respect, each case organization chose its own way to structure its widely varying range of supply. On the basis of our empirical data, no one best way for categorizing long-term care supply can be identified. However, our study suggests that the systematic organization of supply in modules, sub-modules, and subsequent components enabled all case organizations to get a grip on their complex range of supply. Irrespective of the focus chosen, the organization of supply created a transparent structure that provided an orderly presentation of choice options for clients. It might also allow organizations to evaluate their range of supply and more systematically deal with overlap and gaps [19].

Based on our case findings and indicated benefits we would advise providers of long-term care to structure and group their components clearly in order to manage complexity and create transparency in the options provided. This would enable professionals involved in the specification process to relate supply to the multiple and diverse demands of long-term care clients more easily.

### Variation in care service supply

A variety of modular base packages was supplied by the cases. As such, the cases were beginning to exploit analogies among elderly clients. Pre-grouping of components into base packages enables organizations to reduce variability [21,25] with respect to needs and requirements that are similar for all clients. At the same time, it allows organizations to take into account diversity in client demand [21,25] since the packages provide the ability to address directly different types of long-term care clients with services that meet their particular needs. Our data showed that distinctions among base packages can easily be achieved by making different component combinations while drawing from the same set of care service supply. This allows for a more effective and efficient way of working and helps care professionals to directly tune organizational practices to a particular segment of elderly clients [13]. In addition, room is left for activities required by particular client cases since components can be added to or withdrawn from the base package based on individual client needs [8]. Thus, based on our insights, pre-grouping of components based on similarities among all clients and within client segments (e.g., based on health status, constraints faced by clients or the development of constraints and diseases over time) seems to form a largely client-centered starting point for care package specification on the level of individual clients since it explicitly recognizes that clients differ in their needs and preferences [60].

Following our case insights, we would recommend providers of long-term care to mix and match their total range of service supply in such a way that base packages come into existence. Key components in the various base packages should be kept stable according to needs that are common to all elderly clients, and according to needs and wants of segments of elderly clients to ensure quality and efficiency gains. At the same time flexibility should be allowed to take into account individual requirements as well as changes over time.

### Client interaction in modular processes

To specify a client's needs and requirements for long-term care, and subsequently configure a modular service package, the case organizations developed a process that both stimulated and managed client interaction. Our data revealed postponement practices in this process in the sense that customization activities occurred only later in the specification process. Execution of common-to-all activities early in the process and postponement of customization activities allows for effective customization [37,61]. Moreover, while the process evolved, the intensity of client contact increased so that a thorough understanding of the elderly client was created. Especially when client requirements are ambiguous and unclear, it is important to postpone customization activities until after detailed client specifications are received [37]. This is very applicable to long-term care provision, where holistic understanding of the client is vital [62]. Taken altogether, our study suggests that increasing intensity of interaction and postponement of customization activities enabled the case organizations to take into account multiple and changing client needs, and characterize and manage cause-and-effect relations as well as possible interactions between content components, which is necessary for modularity to work [8].

Based on our case insights, we would advise providers of long-term care to review and redesign their processes for determining what care and service components can and will be provided to individual clients. In this, guidance is provided by the postponement principle, i.e. breaking up a process into clearly distinguishable activities, moving forward those activities that have to be executed for all clients in the same manner and postponing those activities that are specific to individuals. In addition, the degree of intensity of client contact should gradually increase over the course of the process. As such, processes can be developed that allow for efficiency as well as for customizing the care offering to the multiple and diverse needs and want for long-term care.

### Joint delivery through people

Despite the operational arrangement of both care and service supply and the specification process, partitions between teams, departments, and professional specialties were highly prevalent in all case organizations. Instead of determining with the elderly client how the organization as a whole could assist this client, a professional attitude that looked at what a particular (sub) specialty could do to resolve a particular problem or relieve a certain constraint from which a client suffered seemed to dominate. Problems accompanying this fragmented approach to care provision are, among others, inefficiency, ineffectiveness, commoditization, and de-professionalization [9]. Based on our case results, we posit that the ability to jointly deliver care and services that provide an answer to the multiple and changing constraints of long-term care clients is at least partly influenced by and dependent upon people. Professionals have to be aware of what is available within their organization and they have to be able and willing to cooperate with and act upon the total knowledge base of their organization.

One way to bring people together, from a modularity point of view, could be to render the idea of mixing and matching into the arrangement of people. A modular arrangement of people would imply that professionals are assigned to clearly defined teams. Each team is a collection of professionals that are competent and qualified to provide one or more care and/or service modules. To enable various departments or organizations to work together it is essential that people from different teams can be rearranged flexibly into modular entities [37]. As such, multi-disciplinary entities can be mixed and matched that have distinctive capabilities, responsibilities, and resources in line with the requirements of a particular client (group) [63]. Because various professionals from different (sub) specialties are working closely together in care and service provision to one long-term care client, the client's needs and requirements can be met more fully and holistically. Professionals are able to act on their specific parts but at the same time adequately appreciate their relation to the evolving whole of client needs [9]. At the same time, clear boundaries and specialty practice of individual professionals allows each professional to perfect his or her skills for care and service provision [22].

Modularity principles and practices applied to the organizational arrangement of people, thus, might be one possible way to break through the provision of partitioned and fragmented care. Integrative solutions can be achieved by purposefully configuring and reconfiguring multidisciplinary work teams that enable professionals to answer the multiple and changing needs of long-term care clients together.

### Reflections

This study has been used to indentify modularity aspects used in current working practices in long-term care provision. The qualitative methodology used allowed us to start building insights and theory on modularity in the field of long term care. The results presented rich descriptions of the case organizations' working practices and to care providers gave insight into which aspects they should consider in order to optimize long-term care provision towards the objective of demand-based care. In addition, this study might serve as a starting point for academics who aim to further develop and test theory on long-term care modularity by means of both qualitative and quantitative methods. Furthermore, we were able to look at care practices through a novel lens and give clues to both health researchers and care practitioners on how to overcome organizational issues faced from a fresh perspective. To ensure that we stayed as close as possible to both the care and modularity perspectives taken in the current study and to take care of a balanced blending of theory and empirical insights from both perspectives, this research was designed around a multidisciplinary research team. Two researchers have their background in the field of operations management (CB and BM), two researchers have their background in health care research (KL and JS) and all researchers were outsiders to the case organizations.

Nevertheless, there were some weaknesses and difficulties in our study. We employed various tactics to sustain and support the validity of our findings such as data triangulation, member checks, and the use of multiple coders. Still, data collection and analysis were complicated by the fact that the interviewees normally would not express themselves using modular concepts nor think about their products and processes as being modular, even though all interviewees recognized the concepts of modularity in their day-to-day practices. We deemed it necessary to provide the interviewees with only limited background knowledge to make sure that they would give us information based on actual working processes and procedures rather than statements colored by explanations of modularity theory. As such, the modularity terms and labels used in this study are our well considered interpretations of sector-related denominations as phrased by the interviewees. Still, it should be noted that the difficulties faced may be the cause of discrepancies between the researchers' interpretation of the data and the expressions used by interviewees.

Furthermore, we are aware that we have left out many of the social aspects related to demand-based care provision. Clients appreciate subtle aspects that go beyond the technical contents of care and the appropriate combination of care and service components such as personalization of interactions and treatment [62,64]. Although we recognize the importance of the emotional and relational characteristics of demand-based care, we chose to focus only on the operational characteristics of demand-based care. As such, we opened up a working language and point of departure for elaborating demand-based concepts in a long-term care setting.

## Conclusions

Based on empirical case research, this study aimed to investigate the potential of modularity in moving the sector of long-term care for the elderly towards the objective of demand-based care provision. The cases provided us with a rich empirical understanding of a new phenomenon in the field of long-term care and its relation to demand-based care provision and allowed us to start building insights and theory in relation to demand-based care provision.

The insights that have been presented in this paper stem from ideas that were developed in manufacturing and service settings, where thorough thinking about the design of processes and systems has greatly improved both quality and efficiency [65]. By looking upon the sector for long-term care from a modularity perspective, we were able to show how different modularity practices can contribute to the creation of a system providing demand-based care.

Making use of product modularity insights enables organizations to accommodate diverse client demands in different ways and provide clients with choice options and variation. In addition, modularity provides guidance during needs assessment and subsequent package specification since it allows organizations to both stimulate and manage client involvement in the specification process. We briefly elaborated on how modularity could further assist long-term care providers in joint provision of care and services when applied to the arrangement of professionals.

The trend towards demand-based and client-centered approaches to care provision is topical all over the developed world and in many fields and sub-sectors of health care systems [e.g. [3,5]]. Therefore, all kinds of healthcare organizations might benefit from the insights presented in this paper in order to approach the operational implications of demand-based care. Adequate setup of an organization's supply, as well as its specification phase activities, is indispensible for providers in every healthcare field who aim to do better in terms of quality and efficiency. Therefore, recognition of the potential of modularity is an important first step in elaborating demand-based care and service provision at the operational level.

## Competing interests

The authors declare that they have no competing interests.

## Authors' contributions

CdB designed and carried out the study, analyzed the data, produced the first draft and revised the manuscript. KL and BM assisted in study design and data analysis. KL, BM and JS revised the manuscript. All authors read and approved the final manuscript.

## Pre-publication history

The pre-publication history for this paper can be accessed here:

http://www.biomedcentral.com/1472-6963/10/278/prepub

## Supplementary Material

Additional file 1 **Supplemental tables**. Word DOC containing Table S1 and Table S2. Click here for file
