# Supplementary material for: Improving long-term care provision: towards demand-based care by means of modularity
Source: BMC Health Serv Res. 2010 Sep 21;10:278. doi: 10.1186/1472-6963-10-278 (PMC2955018; doi:10.1186/1472-6963-10-278)
Supplement: Additional file 1 — Supplemental tables. Word DOC containing Table S1 and Table S2. [file 1472-6963-10-278-S1.DOC]

**Table S1 - Arrangement of choice options: different approaches to a single aim**

All cases had undertaken the challenge to manage their wide range of service supply. Each did so in its own manner, depending on the starting point taken and outcomes aimed for.

**Case 1** decided to functionally organize its range of supply and established three broad modules, that in turn were further divided into sub-modules: ‘care’ (domestic care, nursing care, treatment, health, and comfort services etc), ‘welfare and social support’ (information and advice, social care, accompaniment, etc.) and ‘housing’ (housing adaptations, cleaning services, etc.). Each sub-module, in turn, contained a number of components. For example, the sub-module ‘domestic care’ contained components such as ‘dusting’, ‘mopping’, ‘window washing’, ‘cleaning the bathroom’, ‘cleaning the kitchen’, ‘vacuum cleaning’, etc. Furthermore, broad categories of elderly clients were identified and connected to the various (sub) modules of supply to give direction to both clients and professionals in the selection of appropriate care and service components.

**Case 2** took the day-to-day life of elderly clients as a starting point and the total range of care and service supply was reorganized into modules according to various aspects of daily living. Modules (and sub-modules) were: ‘comfort’ (e.g. pedicure, laundry service, meal service), ‘coziness’ (e.g. social activities, sports activities, creative activities), ‘safety’ (e.g. alarm service, aid devices, night care), ‘peace and balance’ (e.g. day care, day nursing), ‘helping hand’ (e.g. domestic care, nursing care, personal care), and ‘road to recovery’ (e.g. specialist nursing care, physiotherapy, dietetics). Components were grouped under each (sub) module. Moreover, based on the degree to which elderly clients were able to take care of themselves, various client segments were identified. Client segments are clusters of people who require a specific but common combination of care and related services to answer a particular need [13]. Based on needs commonalities within segments of the elderly, component variations were pre-grouped in base packages. These base packages served as stabilized formats based on which individual care and service packages could be further specified and fine-tuned.

**Case 3** decided to functionally organize its range of supply and established broad modules based on four main aspects of living: care, safety, welfare, and housing, under which components were grouped. Moreover, based on the various long-term constraints or diseases from which elderly clients might suffer, components were pre-grouped into base packages. For example, components were pre-grouped for elderly clients suffering from diabetes, COPD, incontinence, motor conditions, etc. In addition, the course of development of each constraint or disease over time was taken into account in the base packages as well. As such, all current and potential future issues that would be likely to occur for a particular constraint or disease, such as diabetes or cancer, were described in a way that clarified what components could or should be changed over time. The base packages served as dynamic formats based on which individual care and service packages could be specified and fine-tuned over time.

**Case 4** had as its main aim to keep the range of supply recognizable for its care professionals and from there be able to deliver good quality care to elderly clients. They therefore chose to reorganize care and service components according to the qualifications of various nursing levels. Thus nursing levels were used as main modules, and included components that each nursing level was allowed to provide. In addition, to ensure that each elderly client was diagnosed and served in a holistic way modular working teams were created, consisting of professionals from various functional areas. These professionals could be easily gathered around a client based on the client’s needs and requirements to decide on the appropriate care and services to be provided. Working teams could be easily dissolved and regrouped. To facilitate the modular working teams, key nurses were appointed to ensure easy access to the collection of components for which each nurse layer was qualified. Moreover, all professionals were stationed in the same building that was specially designed for easy communication and information exchange.

**Table S2 - The process of needs assessment and package specification**

To meet the demands of a given elderly client, certain tasks were performed in each case organization to specify needs and requirements and compose the required care and service package accordingly. In this specification process, professionals of the respective cases determined together with an elderly client what ‘should, can and will be delivered’. Irrespective of some differences in the operational set-up of the specification process, in all cases we observed that specification took place partly before the start of care and service delivery but also partly coincided with it. Therefore, we discerned two phases in the specification process that elderly clients passed through in order to create the optimal care and service package. We call these phases the preparatory phase and the on-the-job phase, respectively.

In the preparatory phase, the elderly client approached the organization, often with a range of relatively undefined and generic questions that could concern all aspects of life. To narrow down these questions and make the needs and demands of the client more specific, a generic needs assessment took place. In all cases, needs and requirements were roughly defined and explored by a professional by means of a phone questionnaire. For further needs clarification a house visit was paid during which the elderly client was interviewed and his living environment was observed by means of a check list. Based on the generic assessment the organization was able to construct a preliminary package of relatively standard care and service parts (or roughly adapt the appropriate base package) after which delivery started.

The second phase of needs assessment took place simultaneously with the first period of care and service delivery. In this phase, the elderly client and professional together experienced the delivery of care and service components. This led to an in-depth exploration of the client’s needs, requirements, and particularities with respect to each need identified and subsequent component(s) provided to meet this need. For example, when an elderly client needed assistance in washing, the time needed was investigated as well as the habits and rituals of the client (e.g. preference for taking a shower or having a bath, whether the client puts on body lotion afterwards, etc.). Based on the detailed needs assessment that took place in close and intensive interaction between elderly client and professional, various dimensions of the preliminary package could be changed and adapted. Adaptations concerned aspects related to the content of components. For example, based on clients’ state of health and preferences, the generic component ‘domestic care’ might for one client comprise the execution of all household chores (both heavy tasks as mopping and window washing and light tasks such as dusting) whereas for another client it would include assistance with heavy tasks only. In addition, adaptations were related to component execution (time of delivery, frequency, duration, and intensity) that could be varied among elderly clients.

After a certain period all needs and preferences of an elderly client were known and the care package could be finalized. Over time, adjustments in the care and service package would probably be necessary because of changing client circumstances. The process of package adaptation, however, is beyond the scope of this paper. To illustrate the above, figure 2 depicts the sequential phases of the specification process.
